# As to Canada

**Published:** 1883-11

**Authors:** 


AS TO CANADA.

Time was when the average citizen of the United States was supposed to spend his spare hours in devising plans for the annexation of Canada to his own country. As a matter of fact, he now occupies himself more profitably upon more congenial subjects; and gives about as much attention to the subject as to the slightly confused problems presented by the presidential succession in Mexico. He has indeed, perhaps, on occasion, run the gauntlet of the custom house officials on the dividing line between the two countries; submitted without a murmur to the rate of discount with which his notes and silver were accepted; looked out drearily upon the dismal panorama of wilderness and villages visible from the windows of the railway carriage; gazed with something like astonishment upon the helmeted men and no less oddly-dressed women of the Dominion, playing with sad expression of countenance their fond part in the small theater where royalty is mimicked; and then returned to Boston, New York, Philadelphia or Chicago, well cured of any delusions respecting the desirability of any more intimate union between the two countries than that which has been provided by nature.

To medical men and matters in Canada, the physicians of the United States have paid but little attention. They have occasionally read with a languid curiosity, references to the quarrels which, in the Dominion as elsewhere, serve to divert the average medical mind from the dull routine of professional toil. They have also known pretty generally that, while Canadian physicians were free to come and settle in all the United States, and to practice in every part of them, without the necessity of surmounting greater barriers than the crude tests here and there provided by some State Board; on the other hand, the graduates of medical schools of the United States were excluded from similar privileges by the intervention of laws which practically forbade emigration thither. Thus much they knew, but they cared little for it. This was too large, that too small a field, to present anything but a contrast in favor of the former to the physician of education and experience.

They also observed a fact of some interest in this connection. Two classes of physicians they had the opportunity of observing in a progress hitherward from the Dominion. The one class, each man with a row of cabalistic letters after his name which only an expert could distinguish from the similar titles of the British Universities, deeming themselves of better medical breeding than the physicians with whom they here came in contact, utterly failed of success as a consequence, and returned in disgust the land from which they came. The other class, accomplished, skillful, and wiser in the wisdom of this world, soon sank their nationality in an American citizenship, and achieved a consequent success which could otherwise have been reached only with difficulty.

Of all Canadian medical facts, however, those which are actually brought into greatest prominence before the profession in this country, are its medical journals. For years we have had the opportunity of glancing through their columns, and on each occasion have been impressed with a feature common to them all in successive years. This feature is the manifest extent to which they seem to be dependent for their maintenance and support upon the medical writers, publishers, and manufacturers of this country.

Look, for example, at their advertisements. We rather expect to find the average cross-roads medical journal in the land of the Yankees, eking out a precarious existence by noticing the success of the Messrs. Brown in sugar-coating their pills, and of the Messrs. Jones in the sale of their vaunted and valuable "Pathine." It is true that the utterances of its editor and the outgivings of his correspondents do not possess the authority which under more favorable circumstances they might enjoy. But what of that? Are they not all our brethren? How, under heaven, could the Court and Nobility of the medical world set the fashions for a whole continent, if these little sheets did not waft their wise sayings and doings all over the face of it?

Ought we not, however, to look for greater things from the men who write after their names, M.B.; F.R.C.S.; L.R.C.S.; L.R.C.P.; F.O.S., and all the other letters of the bewildering alphabet of true greatness? Have the Yankee pill man and the "American" extract fellow, succeeded in subsidizing also them and their Press?

Here, for example, is the latest issue of the *Canada Medical Lancet*, our very respectable contemporary, which claims to have "the largest circulation of any medical journal in Canada." A

careful examination of the advertising pages furnishes us with the following items:

Thirty-six advertisements appear of articles supplied by the United States. Most of these notices are large, indeed, the largest printed, filling a three-fourths or entire page; some requiring several pages. Prominent among these are the announcements of our "preparation" men, whose enterprise on this side of the line has stocked the desk of the physician with samples which accumulate more rapidly than the office boy can dispose of them. Five medical schools of the United States, and four of Canada, are also here advertised, the notices of the latter being among the largest "native" advertisements in this issue, suggesting thus that even in the Dominion, the diploma industry is not without its tangible rewards. Here is also a full page bookseller's advertisement, announcing seventeen treatises whose authors or publishers were of this country; three of English authorship and imprint; two not stated. There are fifteen small "native" advertisements, being for the most part notices of practice fors ale, medical cards, and minor announcements. Finally, there are six advertisements of articles furnished neither here nor in Canada.

Of the Selected Articles in this number, five are credited to this country, two to Great Britain, and the original source of one is not named.

There are eleven book reviews, ten of works of "American" authorship; one only of native growth, being an effort to teach the Canadians "How to Draw a Simple Will."

We have selected this number of this journal at random; and it is a very fair sample of what we are accustomed to see in the columns of our Canadian exchanges, and have so seen for many years past. The evidence is tolerably clear, that three-fourths of all the advertising support of the medical journals published in the Dominion, and probably a larger proportion of all books reviewed by them, are furnished on this side the imaginary line which divides the two countries.

There is food for reflection here, whose digestion we may well leave to our friends in Canada. There are few American physicians who can look inside of a Canadian medical journal and repress a smile. Often, indeed, they are in this way reminded of an incident, lately made public, connected with the transportation of the mails between the two countries. It was discovered, in the course of the international exchanges necessitated by the postal service, that the mail-bags of the United States had so accumulated in Canada that the latter country had found it quite unnecessary to provide any for her own use. And thus it came about that the postal matter there was distributed in mail pouches stamped with the emblem of a nation which Canadians for the most part affect to despise.

